# Supplementary material for: In Vivo Tracking for Oncolytic Adenovirus Interactions with Liver Cells
Source: Biomedicines. 2022 Jul 13;10(7):1697. doi: 10.3390/biomedicines10071697 (PMC9313019; doi:10.3390/biomedicines10071697)
Supplement: Supplementary file 1 [file biomedicines-10-01697-s001.zip › supplementary materials.pdf]

Supplementary materials

In vivo tracking for oncolytic adenovirus interactions with liver cells

Victor A. Naumenko, Daniil A. Vishnevskiy, Aleksei A. Stepanenko, Anastasiia O Sosnovtseva, Anastasiia A Chernysheva, Tatiana O. Abakumova, Marat P. Valikhov, Anastasiia V. Lipatova, Maxim A. Abakumov, Vladimir P. Chekhonin

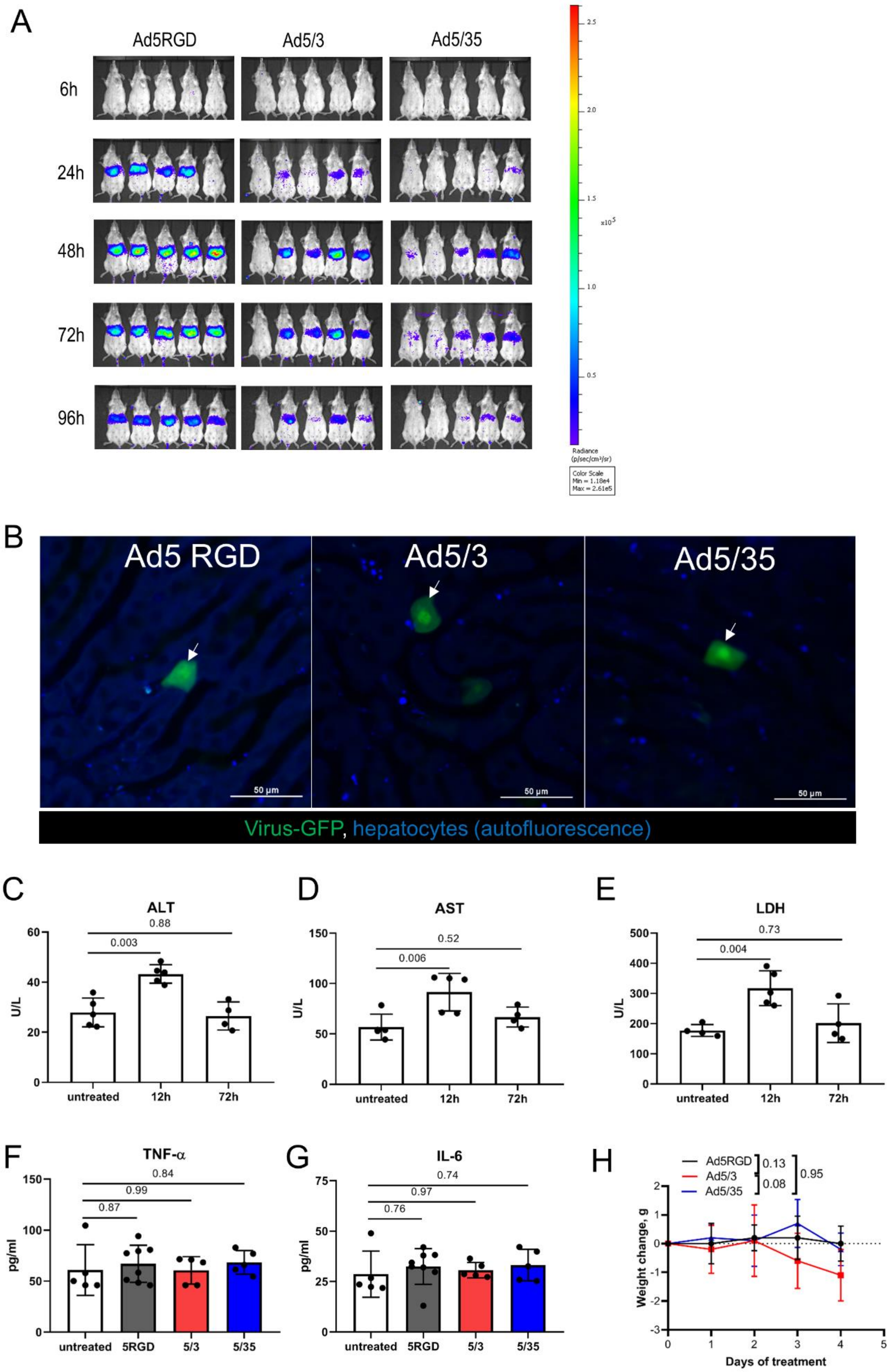

Figure S1. ***In vivo*** **transduction of hepatocytes does not correlate with toxicity. A.** Bioluminescent images for Figure 1C. **B.** Liver intravital imaging at 24 h after i.v. administration

of EGFP-expressing adenoviral vectors (1.6×10^9 IFU). Arrows show transduced hepatocytes. **C-E.** Plasma levels of alanine aminotransferase **(C)**, aspartate aminotransferase **(D)**, and lactate dehydrogenase **(E)** measured at indicated time points after i.v. administration of adenoviral vectors (7×10^8 IFU; mean ± SD; *p*-values are shown on graph; 1-way ANOVA followed by Dunnett's multiple comparisons test). **F-G.** Plasma levels of TNF-α **(F)** and IL-6 **(G)** measured at 12h after i.v. administration of adenoviral vectors (7×10^8 IFU; mean ± SD; *p*-values are shown on graph; 1-way ANOVA followed by Dunnett's multiple comparisons test). **H.** Body weight change measured at indicated time points after i.v. administration of adenoviral vectors (7×10^8 IFU; n=5; mean ± SD; *p*-values are shown on graph; 2-way ANOVA followed by Tukey's multiple comparisons test)

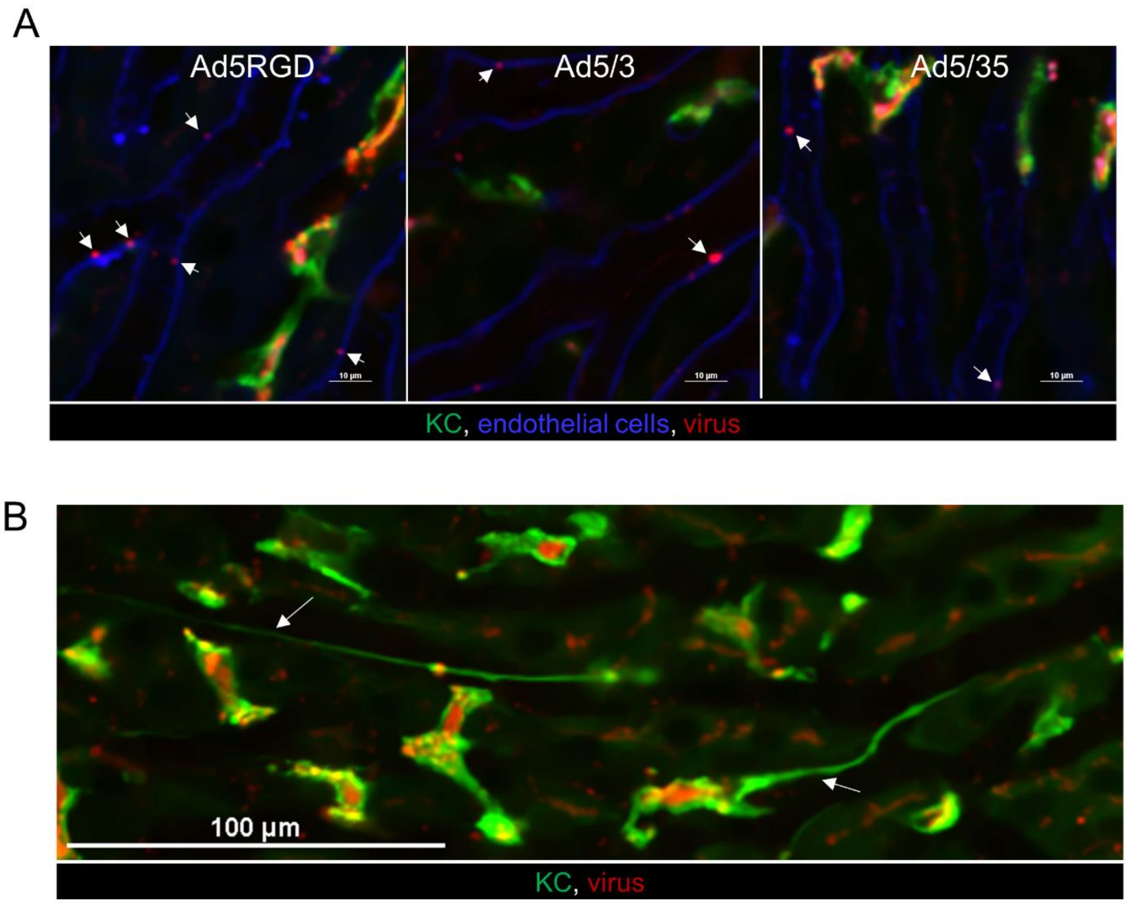

Figure S2. Ad5-RGD and Ad5/3 vectors lead to rapid zeiosis of Kupffer cells. A. Binding of adenoviral vectors to endothelial cells (arrows) immediately after i.v. injection of 10^{10} VP labeled with AF647. **B.** Long-extending protrusion of virus-bound KCs (arrows).

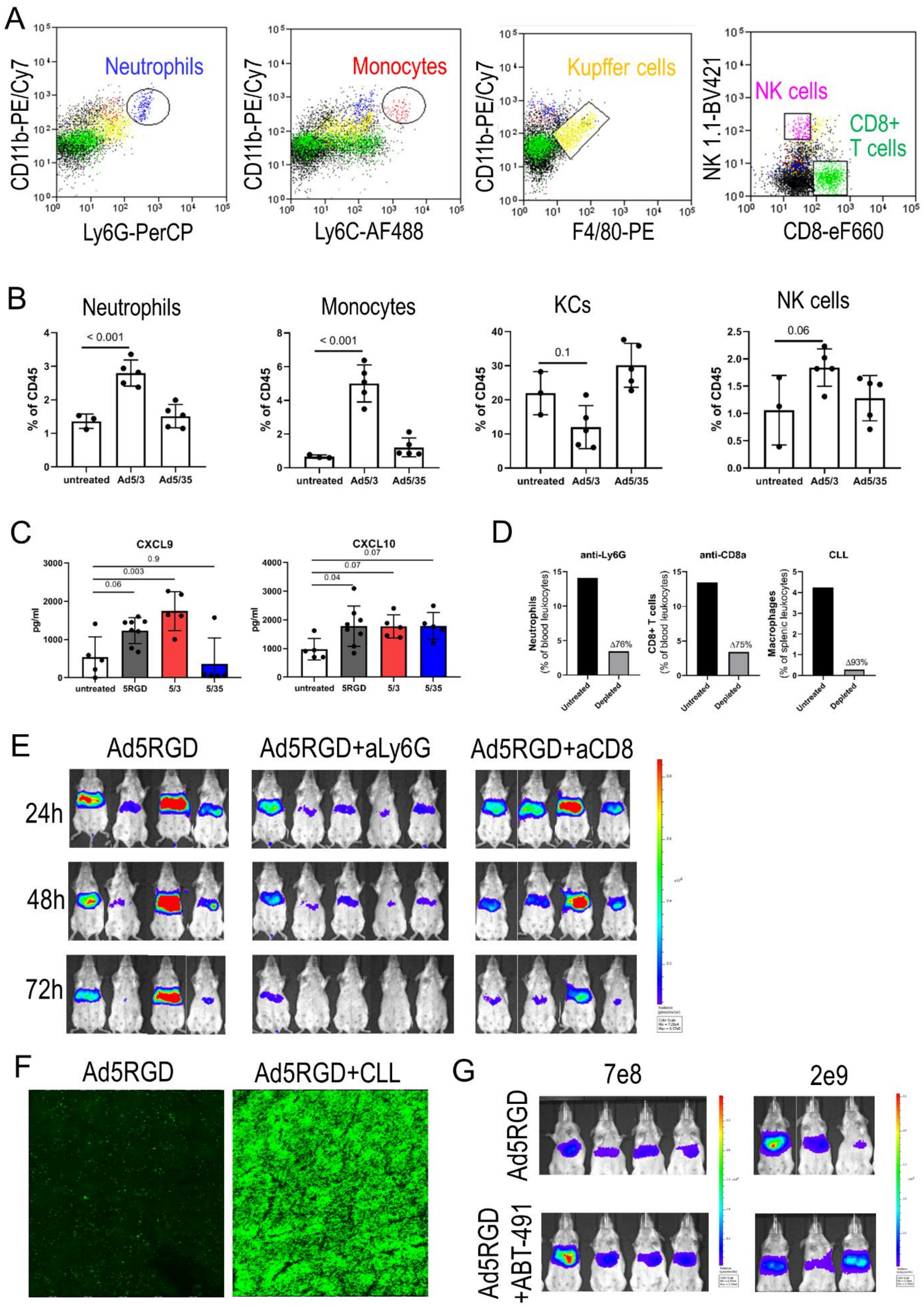

Figure S3. Immune response and clearance of liver infection. A. Gating strategy for detection of liver leukocyte subpopulations by flow cytometry. **B.** Liver leukocyte frequencies at 12 h after

injection of 7×10^8 IFU Ad5/3 and Ad5/35 (flow cytometry; mean ± SD; *p*-values are shown on graph; 1-way ANOVA followed by Dunnett's multiple comparisons test). **C.** Plasma levels of CXCL9 and CXCL10 measured at 12 h after i.v. administration of adenoviral vectors (7×10^8 IFU; mean ± SD; *p*-values are shown on graph; 1-way ANOVA followed by Dunnett's multiple comparisons test). **D.** Efficiency of anti-Ly6G, anti-CD8 antibodies, and clodronate liposomes (CLL) in depleting blood neutrophils, blood CD8+ T cells, and splenic macrophages, respectively. **E.** Bioluminescent images for Figure 3G. **F.** EGFP-expression in the liver 24 h after injection of Ad5-RGD-EGFP (1.6×10^9 IFU) into mice with or without CLL pretreatment **G.** Bioluminescent images for Figure 3H showing levels of liver infection ± ABT-491 treatment.

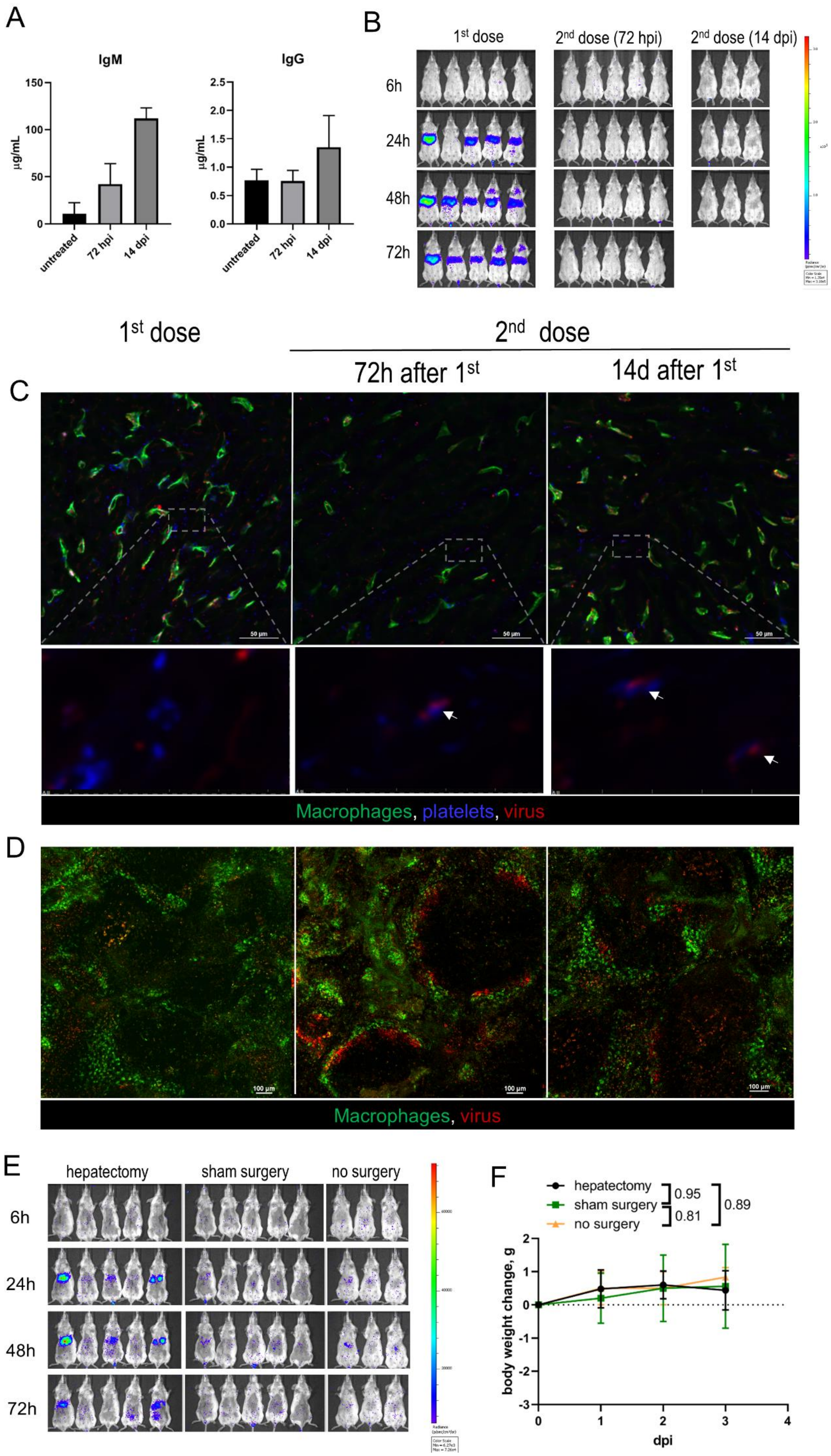

A
IgM
IgG
µg/mL
untreated
72 hpi
14 dpi
B
1st dose
2nd dose (72 hpi)
2nd dose (14 dpi)
6h
24h
48h
72h
1st dose
2nd dose
72h after 1st
14d after 1st
C
50 µm
Macrophages, platelets, virus
D
100 µm
Macrophages, virus
E
hepatectomy
sham surgery
no surgery
F
hepatectomy
sham surgery
no surgery
0.95
0.81
0.89
body weight change, g
dpi

Figure S4. Adenovirus infection and toxicity in preimmunized hosts and in regenerating liver. A. Plasma levels of anti-Ad5-RGD IgM and IgG measured by ELISA at 72 h and 14 days after treatment (7×10^8 IFU; mean ± SD). **B.** Bioluminescent images for Figure 4A. **C.** Representative IVM-images of platelet-virus interactions (arrows) in the liver immediately after delivering of AF647-labeled Ad5-RGD (10^{10} VP) as a first or repeated dose. **D.** Representative *ex vivo* images of spleen 90 min after i.v. administration of AF647-labeled Ad5-RGD in naïve and virus-pretreated mice. **E.** Bioluminescent images for Figure 4F. **F.** Body weight change measured at indicated time points after i.v. administration of 2×10^8 IFU Ad5-RGD in hepatectomized mice (n=5), sham-control group (n=5) and mice with no preceding surgery (n=3; mean ± SD; *p*-values are shown on graph; 2-way ANOVA followed by Tukey's multiple comparisons test).

Supplementary movies

Movie 1. Uptake of adenoviral vectors by Kupffer cells.

Green – Kupffer cells, blue – endothelial cells, red – virus.

Movie 2. Zeiosis of virus-bound Kupffer cells.

Green – F4/80, blue – CD11b, red – virus. Arrows point to membrane blebbing

Movie 3. Virus trafficking in apoptotic protrusion.

Green – Kupffer cells, blue – neutrophils, red – virus. Arrow follows virus trafficking within cell

Movie 4. Virus release in membrane-associated vesicle.

Green – F4/80, blue – CD11b, red – virus. Arrow follows virus release.

Movie 5. Neutrophils participate in clearance of virus-loaded Kupffer cells

Part I. Neutrophils sweep out the virus-containing Kupffer cells. Green – Kupffer cells, blue – neutrophils, red – virus. Arrow points to neutrophil-Kupffer cell interaction.

Part II. Neutrophils transfer virions and fragments of Kupffer cells. Green – Kupffer cells, blue – neutrophils, red – virus. Arrow follows migrating neutrophil with the virus.

Movie 6. Impaired permeability of Kupffer cells following Ad5-RGD and Ad5/3 injection.

Green – Kupffer cells, red – propidium iodide.

Movie 7. CD8+ T cells long-term interactions with Ad-transduced hepatocytes.

Part I. Green – virus-EGFP, blue – Ly6G, red – CD11b, gray – CD8+ T cells. Arrow points to CD8+T cell contact with hepatocyte

Part II. Green – virus-EGFP, blue – endothelial cells, red – CD49b, gray – CD8+ T cells. Arrows point to CD8+T cell contacts with hepatocytes

Movie 8. Apoptosis of Ad-transduced hepatocytes.

Green – virus-EGFP. Arrow points to hepatocyte undergoing apoptosis.

Movie 9. Uptake of Ad5-RGD by Kupffer cells in naïve and virus-pretreated mice

Green – Kupffer cells, blue – platelets, red – virus.
